# Fraud Detection Protocol for Web-Based Research Among Men Who Have Sex With Men: Development and Descriptive Evaluation

**DOI:** 10.2196/12344

**Published:** 2019-02-04

**Authors:** April M Ballard, Trey Cardwell, April M Young

**Affiliations:** 1 Department of Epidemiology College of Public Health University of Kentucky Lexington, KY United States; 2 Department of Environmental Health Emory University Atlanta, GA United States; 3 Center on Drug and Alcohol Research University of Kentucky Lexington, KY United States

**Keywords:** fraud, HIV, mobile phone, MSM, Web-based research, questionnaires, technology, validity, Web-based methodology

## Abstract

**Background:**

Internet is becoming an increasingly common tool for survey research, particularly among “hidden” or vulnerable populations, such as men who have sex with men (MSM). Web-based research has many advantages for participants and researchers, but fraud can present a significant threat to data integrity.

**Objective:**

The purpose of this analysis was to evaluate fraud detection strategies in a Web-based survey of young MSM and describe new protocols to improve fraud detection in Web-based survey research.

**Methods:**

This study involved a cross-sectional Web-based survey that examined individual- and network-level risk factors for HIV transmission and substance use among young MSM residing in 15 counties in Central Kentucky. Each survey entry, which was at least 50% complete, was evaluated by the study staff for fraud using an algorithm involving 8 criteria based on a combination of geolocation data, survey data, and personal information. Entries were classified as fraudulent, potentially fraudulent, or valid. Descriptive analyses were performed to describe each fraud detection criterion among entries.

**Results:**

Of the 414 survey entries, the final categorization resulted in 119 (28.7%) entries identified as fraud, 42 (10.1%) as potential fraud, and 253 (61.1%) as valid. Geolocation outside of the study area (164/414, 39.6%) was the most frequently violated criterion. However, 33.3% (82/246) of the entries that had ineligible geolocations belonged to participants who were in eligible locations (as verified by their request to mail payment to an address within the study area or participation at a local event). The second most frequently violated criterion was an invalid phone number (94/414, 22.7%), followed by mismatching names within an entry (43/414, 10.4%) and unusual email addresses (37/414, 8.9%). Less than 5% (18/414) of the entries had some combination of personal information items matching that of a previous entry.

**Conclusions:**

This study suggests that researchers conducting Web-based surveys of MSM should be vigilant about the potential for fraud. Researchers should have a fraud detection algorithm in place prior to data collection and should not rely on the Internet Protocol (IP) address or geolocation alone, but should rather use a combination of indicators.

## Introduction

The internet is becoming an increasingly common tool for survey research [1-8]. Online instruments present opportunities to recruit “hidden” or vulnerable populations that have previously been difficult to reach, including men who have sex with men (MSM), people who use drugs, and transgender people [2,9-13]. Internet-based research allows for anonymity and decreases barriers for participants. The confidentiality and distance from the researcher provided with Web-based data collection creates a space for participants, especially those who are part of a stigmatized population, to provide information honestly and with less apprehension [2,4,9,14,15]. Furthermore, Web-based methods may be more appropriate and effective in reaching communities that are highly active on the Web, such as MSM who use social and sexual networking websites and dating and sexual mobile phone apps [2]. Internet as a data collection tool also allows researchers to reach geographically dispersed populations [4,9] and minimizes cost to researchers, providing an effective, cost-efficient method to recruit and collect data from hard-to-reach populations [2,3,7,8,14-16].

However, the anonymity of internet-based research that serves as an advantage for data collection also poses challenges that portend data quality and validity. Surveying participants in person allows investigators to better enforce inclusion criteria and prevent duplicate enrollment [2,3,7,9,14,15,17], while the process of avoiding fraudulent enrollment in Web-based research is more difficult. Previous research has focused on 2 different types of invalid entries as follows: (1) ineligible participants who misrepresent themselves to fit eligibility criteria to profit from compensation (ie, misrepresentation fraud) and (2) eligible participants who participate more than once without malefic intent or to receive additional compensation (ie, duplicate fraud) [2,4,7,9,13-19]. The confidentiality and anonymity of Web-based research make it difficult to prevent such entries, posing a threat to data integrity if appropriate data quality protocols are not in place [4,7,13-15,17-20]. Bots and smart software that have the capability to produce human-like data with unique Internet Protocol (IP) addresses can also lead to invalid or fraudulent data [21]. Computer program (ie, bots) and human fraud must both, therefore, be considered in fraud identification strategies. As others have noted [1,2], increasing use of Web-based methodology warrants the development of standards and expectations for Web-based research to ensure data validity and accurate result reporting.

Previous research has focused on the use of a handful of characteristics that can be used to identify fraud (eg, IP address; personal information such as name, address, and email address; response patterns; and timestamps) [2,3,7,9,13-15,17,18,20,22]. However, technology is rapidly advancing, as is the savviness of its users, and approaches previously used to prevent and detect fraud may now lack utility or need to be supplemented with other techniques [2]. For example, the development and increasing use of network devices and smartphones have made it more difficult to utilize IP addresses as they can be masked both intentionally and unintentionally by users [23-26]. The purpose of this analysis was to evaluate fraud detection strategies in a Web-based survey of young MSM and describe new protocols to improve fraud detection in Web-based survey research.

## Methods

### Study Design

The Men’s Health Study was a cross-sectional survey that examined individual- and network-level risk factors for HIV transmission and substance use among young MSM residing in 15 counties in Central Kentucky (total population size, 743,119) [27]. The eligibility criteria included being aged 18-34 years, being biologically male, having engaged in anal sex with another man during the past 6 months, and residing in Central Kentucky. Qualtrics [28], a Web-based survey service, was used to create a Web-based eligibility screening assessment, consent form, and behavioral and demographic survey. A link to the Qualtrics survey was posted on the study website hosted by WordPress [29], which also described the monetary incentive, the process for completing the screening assessment, consent form, and survey, and provided community resources and contact information for the research staff. Based on pilot-testing among the staff, the screening, consent, and survey were anticipated to require a maximum of 45 minutes to complete, and the survey was anticipated to take between 5 and 30 minutes to complete depending on responses and subsequent skip patterns.

Participants were recruited from February to July 2018 utilizing flyers containing the Web address and a quick response code for the study’s website. The flyers were posted in local lesbian, gay, bisexual, transgender, and queer (LGBTQ) venues (eg, bars, adult entertainment stores, and health clinics) and on social media via a study-specific page and young adult LGBTQ groups. Staff also set up booths at 2 local LGBTQ events to disseminate flyers; at one event, the booth allowed space for participants to take the survey and be reimbursed on site. Recruitment also involved peer referral. During the survey and informed consent form, participants were informed that they were allowed to refer up to 3 peers to the study and receive US $10 per eligible referral who completed the survey. Upon completion of the survey, participants received a message from the staff that contained the survey URL and a referral code number and reminded them that they could earn up to US $30 for peer referrals; the former allowed the staff to link participants to people they refer. Participants could choose to receive this message from the staff via a short message service (SMS) text message or email, and provided their phone number or email address accordingly.

Participants who completed the survey had the option of declining an incentive or receiving a US $25 e-gift card or mailed payment and provided details either on their email address or mailing address. Participants were informed that the same method of delivery chosen for their US $25 survey incentive would be used for their peer referral incentives. Those who were recruited at local LGBTQ events were also given the option to receive their US $25 incentive in-person as payment and, then, were asked if they would like to receive their peer referral incentives as an e-gift card or mailed payment. All procedures were approved by the University of Kentucky’s Institutional Review Board.

### Fraud Prevention Strategies

Prior to launching the Web-based survey, 4 strategies were implemented to prevent fraud. First, the informed consent form stated that participants should not take the survey more than once, and if this occurred, incentives would not be received more than once. Second, participants could not enter their email or mailing address to receive compensation until the very end of the survey, making the process to receive incentives more time-consuming in an effort to deter fraud. Third, to prevent participants from taking the survey more than once, either as a duplicate entry or to try to determine the eligibility criteria and subsequently misrepresent themselves to meet those criteria, the “prevent ballot box stuffing” option in Qualtrics was activated. This option places a cookie on the browser when a response is submitted so that if the survey link is clicked on again from the same device and browser, Qualtrics would detect the cookie and prevent the person from entering the survey [30]. Fourth, an option was also activated in Qualtrics to prevent indexing, which blocks search engines from finding the survey and presenting it in search results [30], thereby reducing the likelihood of fraudulent or inapposite participation. Of note, validation using GeoIP location, an estimate of a person’s location based on the IP address, was not used to block survey access because GeoIP location is not always accurate. Smartphones, remote access tools (eg, virtual private network), and network address translators (NATs) used by organizations or companies that assign public addresses to a computer inside a private network for security purposes can result in less accurate or concealed GeoIP estimates [23-26]. In addition, the survey did not use a tool like a Completely Automated Public Turing Test to tell Computers and Humans Apart, which could have reduced fraud by requiring respondents to verify that they were not robots [30]. However, previous research has demonstrated that Completely Automated Public Turing Test to tell Computers and Humans Apart tool verification is not failproof and can be passed despite an entry being invalid or fraud [21].

### Fraud Criteria and Detection Strategies

**Figure 1:**
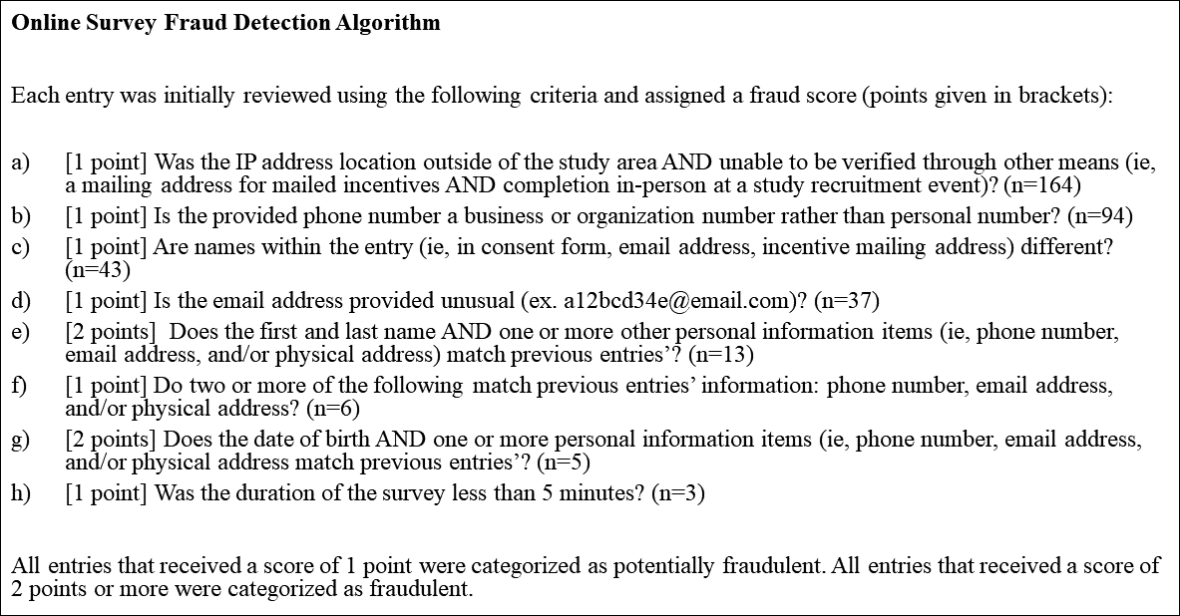
Web-based survey fraud detection algorithm.

Each survey that was at least 50% complete was manually checked by the study staff utilizing an extensive protocol (Figure 1) to detect fraud based on 8 criteria (described below). A point system was used with each criterion having an assigned point value of 1 or 2, and the total was used to classify surveys as fraudulent, potentially fraudulent, or valid. Data characteristics that were considered to possibly indicate fraud but could also have a reasonable justification were assigned 1 point so that they could be flagged as suspicious and confirmed through correspondence with participants. Data characteristics that were considered to be strongly and independently indicative of fraud were assigned 2 points. A 2-point threshold was, therefore, used for classification as fraud. The measures listed below were used in the fraud detection algorithm.

#### Geolocation

The survey software logged approximate latitude and longitude locations for participants by comparing IP addresses to a location database [23,24,30]. These data are typically accurate to the city level within the United States. Outside of the United States, data are only accurate at the country level [30].

Geolocation outside of the study area was valuable for *assisting* with fraud detection but could not be used as a stand-alone fraud detection mechanism because only an external IP address is displayed when a device is connected to a virtual private network or NAT. This causes all devices to have identical IP addresses and geolocation [24,26]. Additionally, research staff found that surveys they knew to be local and valid (ie, those completed at local LGBTQ events) would frequently have geolocations outside of the study area, sometimes even outside of the state of Kentucky. Surveys at LGBTQ events were completed on smartphones, which can display different IP addresses within minutes because of network proxies within the carrier’s network. This results in inaccurate geolocation based on IP addresses [25].

Geolocation based on IP addresses was, therefore, not used in isolation as a fraud detection mechanism but was considered in the context of other indicators of location. In-person recruitment events and mailing address for those who requested payment incentives allowed the study staff to verify the location of participants. If the survey was completed at an in-person event or if the incentive was requested to be mailed to an address in the study area, the IP address geolocation outside of the study area was not used as an indicator of fraud. However, if the IP address geolocation was outside of the study area, the incentive requested was an e-gift card, and the survey was not completed in-person at a recruitment event, then the survey received 1 point toward the fraud detection point total.

#### Phone Number

Participants were asked to provide telephone numbers if they consented to be contacted for future research and as a method to receive a referral code. The staff searched phone numbers on the Web using Whitepages.org to identify instances in which the numbers were invalid or corresponded with local businesses or organizations, in which case, the survey received 1 point [15]. Phone numbers were kept separate from all survey data to ensure participants’ privacy and anonymity.

#### Names

When the survey was initially launched, participants were asked to give their name only if they consented to be contacted for future research or they requested payment through the mail for their incentive. To be able to investigate fraudulent behavior among e-gift card recipients, researchers began requesting participants name when e-gift cards were selected as an incentive. In addition, names were sometimes contained in or associated with email addresses (eg, john.doe@email.com) entered to receive e-gift card incentive, referral code, or community resources. Therefore, names were given up to 3 times—for incentive delivery, through names linked to or contained in email addresses, and in the consent to future research section. This allowed researchers to cross-reference names up to 3 times. If names did not match within a survey, participants were assigned 1 point toward fraud.

#### Email Address

Email addresses were examined to detect potential fraud. As done in a previous study [21], addresses with alternating letters and numbers (eg, a12bcd34e@email.com) were suspected by researchers to be fake email accounts (ie, created by a Bot program or by a human trying to misrepresent themselves). Surveys associated with such email addresses received 1 point toward fraud.

#### Personal Information

Personal information, including name, phone number, date of birth, email address, and mailing address, was compared across surveys to detect fraud. If 2 personal information items (ie, phone number, email address, and mailing address) matched a previously completed survey, the survey received 1 point toward their fraud total. Those with matching information but different names and date of birth could represent phone sharing and cohabitation, so surveys that contained contact information that had been entered previously but different names and dates of birth were contacted by the study staff to verify their identity as a unique participant (further described in *Fraud Categorization* below). Those with 1 or more matching personal information items and the same first and last name as a previous survey received 2 points as this was seen as a definite sign fraud. Additionally, surveys with, at least, one matching personal information item and the same date of birth as a previous survey received 2 points.

#### Survey Duration

Qualtrics collected timestamps for the screening, consent form, and survey sections. For each section, Qualtrics recorded the time each section began and ended. Of note, there was no option to save survey progress, preventing completion across multiple sessions. If the survey was completed in <5 minutes, the entry received 1 point.

### Fraud Categorization

**Figure 2:**
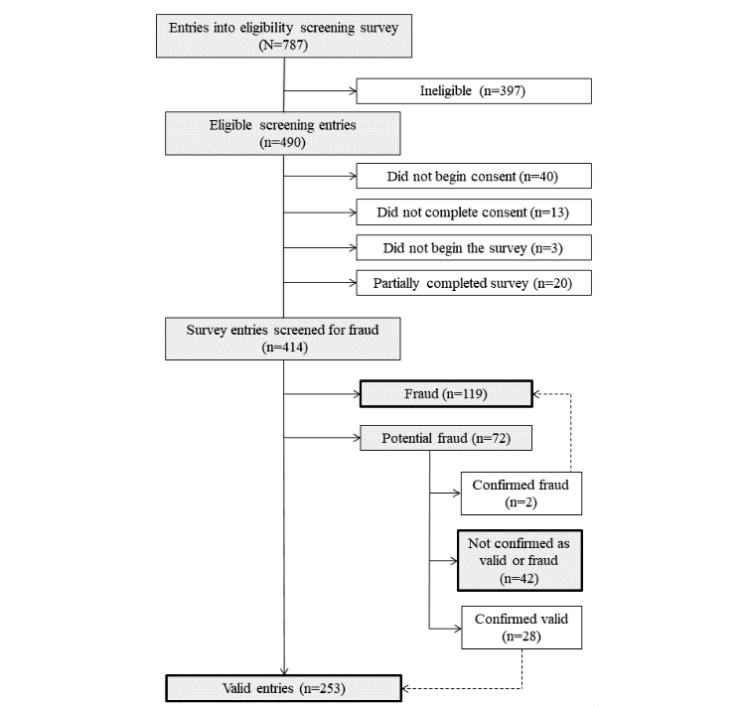
Flowchart of survey entries and fraud categorization.

Figure 2 displays data on survey entries and fraud categorization. Surveys were classified as *fraudulent* if the total entry point value added to ≥2. Depending on what contact information was provided, fraudulent surveys received a phone call, SMS text message, or email stating, “You recently completed a survey for a health study online. However, we detected that your survey entry was fraudulent. If you think this is a mistake, please contact us”, with the intent of deterring further fraudulent behavior. If participants did not respond, their survey was considered invalid and they did not receive compensation. No responses were received from those categorized as fraudulent.

Surveys were classified as *potential fraud* if the total point value added to 1. Potentially fraudulent participants received a phone call, SMS text message, or an email stating, “Thank you for completing the UK Health Study online survey. We have been experiencing fraud in the study and your survey entry has been flagged as suspicious. We sincerely apologize for the inconvenience if this was an error. Please contact us by calling xxx-xxx-xxxx to confirm that you did indeed complete a survey and we will send your $25 incentive.” This message was meant to deter those who were indeed fraudulent but also provide an opportunity for valid submissions to verify legitimacy. They were asked to confirm personal information such as date of birth, mailing address, or email address to validate their survey. If a participant responded and was unable to provide verification, the survey was categorized as fraudulent. If a participant did not respond, the survey remained as potentially fraudulent.

Surveys were classified as *valid* if they met none of the fraud detection criteria or if they were originally classified as potentially fraudulent but contacted study staff to confirm the validity of their survey entry.

### Analyses

Descriptive analyses were performed using SAS software, version 9.4 (SAS Institute; Cary, NC, USA) to describe the number and percentage who violated each fraud detection criterion among those that were classified as fraud, potential fraud, and valid.

## Results

Overall, 62.3% (490/787) of the completed screening questionnaires were eligible. Of 490 who were eligible, 437 (89.2%) completed the consent and 408 (51.2%) completed the survey with an average survey duration of 19 minutes and 41 seconds. In addition, there were 26 partial surveys, of which 18 were missing several measures used for fraud detection and a majority of survey responses and, therefore, were not included in analyses presented below or in the final set of valid responses. Six partial surveys were >50% complete and were included in analyses, making 414 the final number of surveys in need of evaluation for fraud.

Of 414 surveys, 117 (28.3%) were initially categorized as fraud, 72 (17.4%) were categorized as potential fraud, and 225 (54.3%) were considered valid. Of 72 surveys originally classified as potential fraud, 2 participants responded and admitted to living outside of the study area, 42 did not respond, and 28 were able to verify their survey and were reclassified as valid (see Figure 2). Therefore, of the 414 surveys, the final categorization resulted in 119 (28.7%) surveys identified as fraud, 42 (10.1%) as potential fraud, and 253 (61.1%) as valid for the final sample and analyses (see Table 1). Of the 164 who had geolocation outside of the study area and were not mailed payment or recruited at a local event, 23 (14.0%) had locations outside of the United States, 118 (72.0%) in states other than Kentucky, and 23 (14.0%) within Kentucky but outside of the 15-county study area (Table 1).

**Table 1:** The number of surveys that violated the fraud detection measure by category.

Fraud detection measure	Total (n=414), n (%)	Missing, n (%)	Fraud, (n=119)^a^, n (%)	Potential fraud, (n=42)^a^, n (%)	Valid (n=253)^a^, n (%)
Geolocation outside of study area based on IP^b^ address and unable to be confirmed through mailing address or participating at a local event	164 (39.6)	0 (0.0)	109 (91.6)	34 (81)	21 (8)
Phone number was a local business or organization phone number	94 (22.7)	93 (22.5)^c^	94 (79.0)	0 (0.0)	0 (0.0)
Mismatching names within entry	43 (10.4)	71 (17.1)^c^	37 (31.1)	2 (4.8)	4 (1.6)
Unusual email address	37 (8.9)	98 (23.7)^c^	34 (28.6)	1 (2.4)	2 (0.8)
First and last name AND one or more other personal items match other previous entry	13 (3.1)	16 (3.9)^c^	13 (10.9)	0 (0.0)	0 (0.0)
Two or more personal items match other previous entry	6 (1.4)	67 (16.2)^c^	3 (2.5)	3 (7.1)	0 (0.0)
Date of birth AND one or more other personal items match other previous entry	5 (1.2)	5 (1.2)^c^	5 (4.2)	0 (0.0)	0 (0.0)
Survey duration <5 minutes	3 (0.7)	0 (0.0)	0 (0.0)	2 (4.8)	1 (0.4)

^a^Sample size represents final categorizations (ie, reclassification of some that were initially classified as potential fraud as valid or fraudulent based on the verification with participants).

^b^IP: interenet protocol.

^c^Participants were not required to give personal information (ie, phone number, name, email address) if they did not want to be contacted about future research opportunities, declined their incentive, or if they did not complete the section of the survey describing the referral process. If a personal item was missing needed for a measure, the variable was considered missing.

Figure 1 lists each of the 8 fraud detection criterion used with the assigned point value and the number of surveys that fit that criterion. Surveys had the potential to violate 8 criteria that created a point scale from 0 to 10 points. The actually observed score range was 0-5 points. Data showed that 59.4% (246/414) of all entries had geolocation outside of the study area. However, 33.3% (82/246) of the surveys that had ineligible geolocations belonged to participants who *were* in eligible locations (as verified by their request to mail payment to an address within the study area or participation at a local event). The second most frequently violated criterion was an invalid phone number (94/414, 22.7%), followed by mismatching names within a survey (43/414, 10.4%) and unusual email addresses (37/414, 8.9%). Less than 5% (18/414) had some combination of personal information items matching that of a previous survey (ie, criteria e, f, and g in Table 1). Of the 414 surveys, 3 were below the minimum time threshold established by the study staff, completing it in <5 minutes.

Of note, many surveys violated more than one criterion. Of the 164 entries that violated the geolocation criterion, 94 (57.3%) also had invalid phone numbers, 35 (21.3%) had mismatching names within the survey, and 34 (20.7%) had unusual email addresses. Of the 94 surveys with invalid phone numbers, 29 (30.9%) had mismatching names within the survey and 28 (29.7%) had unusual email addresses. In total, 42.3% (175/414) of surveys violated some combination of the geolocation, phone number, mismatching names, or the email address criteria. The detection measure of matching name and personal identifier data were not independently useful in detecting fraud. A total of 10.9% (13/119) surveys had first and last names and, at least, one other personal items that matched that of previous entries, but all of these also violated some other fraud criteria. Similarly, all surveys that had a date of birth (5/119) that matched a previous survey’s date of birth also violated some other fraud criteria.

Of 119 surveys classified as fraudulent, 109 (91.6%) had geolocations outside the study area, 94 (79.0%) provided business or organization phone numbers rather than personal phone numbers, 37 (31.1%) provided mismatching names within the surveys, and 34 (28.6%) had unusual email addresses. In total, 93.3% (111/119) of fraudulent surveys violated at least one of the following criteria: geolocation, phone number, mismatching names, or the email address; 86.6% (103/119) violated at least 2 of those 4 criteria.

IP addresses were not used in the fraud detection algorithm because analyses demonstrated that they would have limited utility. Overall, 23.0% (95/414) of the surveys had an IP address identically matching that of at least one other survey entry; however, 13.7% (13/95) of those with matching IP addresses were surveys completed at in-person local events and were observed by the staff as being unique individuals. Of the remaining 82 surveys, 47 (57.3%) were classified as valid surveys, 12 (14.6%) were potential fraud, and 23 (28.1%) were fraudulent.

## Discussion

### Principal Findings

In this Web-based study of young MSM, a robust fraud detection algorithm involving geolocation, phone number, email address, name, and other personal data revealed that 28.7% (119/414) of all eligible survey entries were fraudulent. The majority of fraudulent surveys involved participants whose IP address had geolocation outside of the study area, invalid phone numbers (ie, local business phone numbers), mismatching names within the survey, and unusual email addresses (ie, a12bcd34e@email.com). These findings are consistent with prior studies’ conclusions that fraud can be pervasive in Web-based research [5,7,9,13-22], but extends previous studies by outlining a step-by-step fraud detection strategy that does not rely on IP address and geolocation, which were identified in this study to have substantial limitations.

Previous studies have used IP address to detect duplicate entries [2,9,13-15,17,19] but this was not used in this study because (1) Qualtrics’ ballot box stuffing prevention feature was used to prevent multiple entries from the same IP addresses and (2) matching IP addresses may not necessarily indicate fraud but occur in instances where >1 eligible participant lives in the same household, shares devices, or is accessing the survey from a communal space (eg, library or other venues with public Wi-Fi access). Furthermore, IP address to identify duplicate entries was not found to be appropriate based on the increasing use of smartphones, remote access tools, and NATs [23-26]. Remote access tools and NATs present the same IP address across users [26] and present challenges to researchers in differentiating between users. On the other hand, smartphones can present different IP addresses within minutes based on the mobile tower the signal is being routed through [26]; this allows individuals to intentionally or unintentionally obscure their IP address and geolocation, therein limiting the usefulness of the measure. In addition, the increasing threat of bots and smart software that produce human-like data with unique IP addresses further limit this measure as a tool to detect fraud [21]. In this study, the majority of repeat IP addresses were valid entries, suggesting that IP address should be used with caution as a measure for fraud detection.

Geolocation linked to IP address to assist in assessing geographic eligibility (ie, residence in the 15-county study area) was used, but in combination with other indicators of fraud because of the inaccuracy of geolocation data that can be introduced by smartphones, remote access tools, NATs, bots, and smart software [21,23-26]. One out of 3 surveys that had ineligible geolocations based on the IP address belonged to participants who *were* in eligible locations, as verified by their request to mail payment to an address within the study area or by the staff observing their participation at a local event. Thus, like IP address, geolocation used for fraud detection should not be used in isolation.

Other measures that were found to be useful in fraud detection were invalid phone numbers, mismatching names within a survey, and unusual email addresses. Invalid phone numbers included a variety of local businesses and organizations, including manufacturing companies, health care organizations, professional associations, and university offices. Surprisingly, names at different parts of the survey (ie, consent form, contact information, and survey incentive information) were entered inconsistently. Distinct email address patterns that emerged among fraudulent surveys included addresses that began with a numeric value, switched between numbers and letters throughout the address, at least, 3 times, and did not appear to include initials or a name. Unexpectedly, the detection measure of matching name and personal identifier data across surveys, which would indicate duplicate entries, was not useful in detecting unique cases of fraud; surveys that violated these criteria also violated other criteria. These findings, however, may only be representative of this sample and a holistic approach should be considered for monitoring and detecting fraud.

Respondents were contacted to verify entries that were suspicious but not blatantly fraudulent and utilized messages tailored to fraud category when recontacting respondents. Similar to previous research among MSM [21], none of the individuals who received the message tailored to fraudulent cases responded, possibly indicating that these were, indeed, fraud. Of those who received the message tailored to potentially fraudulent cases, 38.9% (28/72) responded with reasonable explanations such as using a partner’s phone number or email address or providing a physical address verifying geographic eligibility and were reclassified as valid. Interestingly, 2 of the potentially fraudulent cases that were contacted verified their fraud, explaining that they were indeed outside of the study area and were confused about geographic eligibility criteria. In addition, real-time monitoring of fraud was helpful in deterring subsequent fraud. A majority of fraudulent entries were submitted within the first month of data collection, but the frequency dramatically decreased after staff began calling, SMS text messaging, and emailing targeted messages. Communicating directly with fraudulent participants may help deter continued invalid entries from being submitted. Future studies should consider monitoring fraud in real time and verifying suspicious surveys with respondents if possible.

### Limitations

While the fraud detection algorithm used in the study was robust, there were limitations. For example, participants’ name and phone numbers could be left blank and were left blank by 3.9% (16/414) and 22.5% (93/414) of surveys, respectively. Other data were available to assist in fraud detection in these cases (ie, geolocation, email address, physical address, date of birth, or survey duration); however, those who did not provide name and phone information had a greater likelihood of not being detected as fraudulent. In addition, virtual phone systems that allow for the quick creation of phone numbers may limit the ability to use phone numbers as a fraud detection algorithm, as respondents can create local numbers and enter them in the survey. Furthermore, certain email address providers display names associated with email addresses when messages are sent while others do not; those that display names allowed the staff to better detect name inconsistency within surveys and fraud.

### Conclusions

This study suggests that researchers conducting Web-based surveys of MSM should be vigilant about the potential for fraud. Researchers should have a fraud detection algorithm in place prior to data collection to ensure that (1) data needed for fraud detection are being collected; (2) the informed consent document can describe that surveys will be evaluated for fraud and what the consequences are for incentives; and (3) fraud can be monitored in real time. The latter allows the staff to send messages to those flagged as fraudulent and potentially fraudulent and deter subsequent fraud and avoid misclassification. Importantly, in research involving populations engaged in stigmatized or illegal behavior, researchers should take extra precautions to ensure that messages sent to respondents do not disclose the focus of the study or eligibility criteria in case the message is intercepted by an unintended recipient. In addition, as discussed by Sullivan et al [2], researchers should be mindful that rapid evolutions in technology could impact the utility and relevance of previously published protocols and methodologies. Therefore, continued innovation in fraud prevention and detection for Web-based research will be necessary, as will the development of automated or semiautomated algorithms for detecting and mitigating fraud [2,5,21]. The latter is especially important given that manual approaches, such as the one used in this study, can be labor- and time-intensive and may be difficult to implement in studies with large samples sizes. Of note, manual approaches may continue to be important for small Web-based studies on novel topics [21].

In research involving geographic eligibility criteria (ie, residence in a certain area), automatic algorithms embedded in Web-based survey tools that exclude people based on the geolocation should be used with caution, as geolocations are often inaccurate. Furthermore, in studies with geographic inclusion criteria, the entry of a phone number with a local area code should not be assumed to be a local person, as the entry of local business or organization numbers can be common. Providing the option of receiving an incentive through mailed payment rather than only by e-gift card proved to be a useful tool in fraud detection, not only for the evaluation of whether the mailing address was within the study area for those who opted for mailed payments but also because the staff could deter fraud by contacting respondents who violated multiple fraud criteria and ask for a mailing address for the incentive rather than automatically sending an e-gift card. In addition, the collection of name data in multiple places throughout the survey, as well as email address, phone number, mailing address, and geolocation based on the IP address were, in combination, useful in assisting with fraud detection.

Future research should consider similar algorithms and should publish algorithms used for fraud detection to improve replicability and to benefit the field of online research.

## Abbreviations

MSM: men who have sex with men
IP: Internet Protocol
NAT: network address translator
LGBTQ: lesbian, gay, bisexual, transgender, and queer
SMS: short message service
